# Genetically determined blood lead is associated with reduced renal function amongst individuals with type 2 diabetes mellitus: insight from Mendelian Randomisation

**DOI:** 10.1007/s00109-021-02152-5

**Published:** 2021-10-18

**Authors:** Mohsen Mazidi, Richard Kirwan, Ian G. Davies

**Affiliations:** 1 Department of Twin Research and Genetic Epidemiology, King’s College London, London, UK; 2 School of Biological and Environmental Sciences, Liverpool John Moores University, Liverpool, UK; 3 Research Institute of Sport and Exercise Science, Liverpool John Moores University, Liverpool, UK

**Keywords:** Mendelian randomisation, Blood lead, Chronic kidney disease, Estimated glomerular filtration rate, Diabetes, Nephrology

## Abstract

**Abstract:**

Some observational studies indicate a link between blood lead and kidney function although results remain controversial. In this study, Mendelian randomisation (MR) analysis was applied to obtain unconfounded estimates of the casual association of genetically determined blood lead with estimated glomerular filtration rate (eGFR) and the risk of chronic kidney disease (CKD). Data from the largest genome-wide association studies (GWAS) on blood lead, eGFR and CKD, from predominantly ethnically European populations, were analysed in total, as well as separately in individuals with or without type 2 diabetes mellitus. Inverse variance weighted (IVW) method, weighted median (WM)-based method, MR-Egger, MR-Pleiotropy RESidual Sum and Outlier (PRESSO) as well as the leave-one-out method were applied. In a general population, lifetime blood lead levels had no significant effect on risk of CKD (IVW: *p* = 0.652) and eGFR (IVW: *p* = 0.668). After grouping by type 2 diabetes status (no diabetes vs. diabetes), genetically higher levels of blood lead had a significant negative impact among subjects with type 2 diabetes (IVW = Beta: −0.03416, *p* = 0.0132) but not in subjects without (IVW: *p* = 0.823), with low likelihood of heterogeneity for any estimates (IVW *p* > 0.158). MR-PRESSO did not highlight any outliers. Pleiotropy test, with very negligible intercept and insignificant *p*-value, indicated a low likelihood of pleiotropy for all estimations. The leave-one-out method demonstrated that links were not driven by a single SNP. Our results show, for the first time, that among subjects with type 2 diabetes, higher blood lead levels are potentially related to less favourable renal function. Further studies are needed to confirm our results.

**Key messages:**

What is already known about this subject?

- Chronic kidney disease is associated with unfavourable lifestyle behaviours and conditions such as type 2 diabetes.
- Observational studies have reported an association between blood lead and reduced estimated glomerular filtration rate, but the relationship between lead exposure and renal function remains controversial.

What is the key question?

- Using Mendelian randomisation with data from 5433 individuals from the UK and Australian populations, does genetically determined blood lead have a potentially causal effect on estimated glomerular filtration rate and the risk of chronic kidney disease?

What are the new findings?

- Blood lead levels have a potentially causal effect on reduced renal function in individuals with type 2 diabetes.
- In subjects without diabetes, no such causal relationship was identified.

How might this impact on clinical practice in the foreseeable future?

- This highlights the risk of elevated blood lead, for example, due to environmental exposure, amongst those with type 2 diabetes, which may predispose them to impaired renal function.

**Supplementary Information:**

The online version contains supplementary material available at 10.1007/s00109-021-02152-5.

## Introduction

Chronic kidney disease (CKD) is an age-associated decline in renal function, diagnosed by impaired glomerular filtration rate (GFR) or increased urinary albumin excretion (albuminuria) [1]. Up to 13% of the global population is estimated to suffer some degree of CKD with increasing age associated positively with reduced renal function such that over one-third of those 70 years or older are affected [2, 3]. Chronic kidney disease is a frequently observed comorbidity in multiple cardiometabolic conditions such as type 2 diabetes, hypertension (HT), obesity and cardiovascular disease (CVD) [4–14], considerably adding to the burden of these conditions. As the aforementioned conditions are also components of metabolic syndrome (MetS) [15], it is not surprising that CKD is also frequently associated with this diagnosis [16, 17] which is estimated to affect 20–25% of western populations [18, 19]. Of particular interest in patients with diabetes is the development of diabetic nephropathy, with diabetes being a primary cause of end-stage renal disease in 40–60% of cases, globally [20]. Furthermore, recent research has illustrated that environmental lead exposure may accelerate progressive diabetic nephropathy, and that reductions in body lead levels by chelation therapy can reduce this rate of progression [21].

Similar to MetS and its constituent conditions, the incidence of CKD is associated with unfavourable dietary patterns and lifestyle behaviours such as low levels of physical activity [22–25]. Interestingly, a number of observational studies have found an association between blood lead levels and reduced estimated glomerular filtration rate (eGFR) [26–29], although not to a clinically significant degree, and this finding is not consistently observed [30–32]. Lead exposure may also be associated with a slight hyperfiltration state, which has been found to attenuate the age-related decline in baseline creatinine clearance, a measure of GFR and even increased eGFR [32]. Thus, the relationship between lead exposure and renal function remains controversial, and further investigation is required. While randomised controlled trials (RCTs) are reliable determinants of causal inferences in nutrition science, not all exposure-outcome interactions can be tested. This is due to both a cost and time perspective and also because of ethical considerations brought about by exposing participants to presumed risk factors, in this case, lead.

Alternatively, Mendelian randomisation (MR) analysis uses functional polymorphisms (single nucleotide polymorphisms (SNPs)) associated with specific changes in exposures (e.g. lead) as genetic instruments and can provide unbiased and robust evidence on mechanisms of disease pathogenesis. Thus, MR studies can overcome this shortcoming of RCTs [33]. Unlike conventional observational studies and risk factor–based epidemiology, MR studies are considerably less prone to confounding, residual bias and reverse causation [34]. Therefore, we used MR analysis to obtain unconfounded estimates of the casual association of genetically determined blood levels of lead with renal function.

## Methods

### Study design

A two-sample MR study design was used, in which summary statistics from different genome wide association studies (GWAS) were analysed for the exposures (blood lead) and outcomes (renal function), to estimate the effects of exposure on outcome [35]. Essentially, we applied genetic predictors of blood lead to extensively genotyped case–control studies of renal function (eGFR and the risk of CKD) to obtain estimates of the association of exposure to our clinical outcomes.

### Genetic predictors of exposures

We retrieved summary data for the association between SNPs and circulating lead from the GWAS carried out by the Queensland Institute of Medical Research (QIMR), Australia (*n* = 2603, mean age 47.2 years, 59% women), and from the Avon Longitudinal Study of Parents and Children (ALSPAC) (2830 unrelated mothers, mean age 28.4 years) [36]. Genotyping, quality control and imputation procedures are described elsewhere [36]. If a SNP was unavailable for the outcome GWAS summary statistics, we identified proxy SNPs with a minimum linkage disequilibrium (LD) *r*^2^ = 0.8. We used 13 independent SNPs with a *p*-value < 5 × 10^−6^. To minimize bias in effect estimates induced by correlation between SNPs, we restricted our genetic instrument to independent SNPs not in linkage disequilibrium (*p* = 0.0001). We refer to a set of SNPs that proxy blood lead as “genetic instruments.”

### Genetic predictors of outcomes

Genetic associations with renal function were obtained from the largest available extensively genotyped study based on a meta-analysis (*n* = 133,413 individuals with replication in up to 42,166 individuals) (full details of all studies included are available in the original article) [37]. eGFR was estimated using the four-variable modification of diet in renal disease (MDRD) equation [37]. CKD was defined as eGFR < 60 ml/min/1.73 m^2^. Type 2 diabetes was defined as fasting glucose ≥ 126 mg/dl, antidiabetic drug treatment or by self-reported history. Kidney function and type 2 diabetes were assessed simultaneously.

For GWAS analysis, a centralized analysis plan was applied with each study regressing sex- and age-adjusted residuals of the logarithm of eGFR on SNP dosage levels. Furthermore, logistic regression of CKD was performed on SNP dosage levels adjusting for sex and age. For all traits, adjustment for appropriate study-specific features, such as study site and genetic principal components, was included in the regression and family-based studies appropriately accounted for relatedness. There was no overlap between the exposure sample size and outcome sample size.

### Statistics

We combined the effect of instruments using the inverse variance weighted (IVW) method as implemented in the TwoSampleMR package running under *R*. Heterogeneity was assessed using *Q* value for IVW. To address the potential effect of pleiotropic variants on the final effect estimate, we performed sensitivity analysis including weighted median (WM) and MR-Egger. Sensitivity analysis was conducted using the leave-one-out method to identify instruments that might drive the MR results. The WM estimate provides correct estimates as long as SNPs accounting for ≥ 50% of the weight are valid instruments. Inverse variance is used to weight the variants, and bootstrapping is applied to estimate the CIs [35]. MR-Egger is able to make estimates even under the assumption that all SNPs are invalid instruments, as long as the assumption of instrument strength independent of direct effect (InSIDE) is satisfied [35]. However, the InSIDE assumption cannot be easily verified. Average directional pleiotropy across genetic variants was assessed from the *p* value of the intercept term from MR-Egger [35]. Causal estimates in MR-Egger are less precise than those obtained by using IVW MR [38]. Analysis using MR-Egger has a lower false-positive rate, but a higher false-negative rate, than IVW, i.e. it has a lower statistical power [39].

Heterogeneity between individual genetic variant estimates was assessed by the use of the Q′ heterogeneity statistic [40]. The Q′ statistic uses modified 2nd-order weights that are a derivation of a Taylor series expansion, taking into account the uncertainty in both numerator and denominator of the instrumental variable ratio [40].

To assess the instrumental variable analysis “exclusion-restriction” assumption, we used Ensembl release (http://useast.ensembl.org/index.html) that contains a base of SNP phenotypes and PhenoScanner (Ensembl gives SNP phenotypes, PhenoScanner also gives phenotypes of correlated SNPs.).

### Sensitivity analysis

As sensitivity analysis, we used MR-Egger and MR pleiotropy residual sum and outlier (MR-PRESSO) test [40]. MR-Egger and MR-PRESSO may provide correct estimates as long as the instrument strength independent of direct effect assumption is satisfied [40]. MR-Egger can be imprecise, particularly if the associations for SNPs on exposure are similar, or the number of genetic instruments is low [40]. A non-null MR-Egger intercept suggests that the IVW estimate is invalid. MR-Egger does not explicitly identify outliers. MR-PRESSO detects, and if necessary, corrects for potentially pleiotropic outliers [40]. The MR-PRESSO framework detects effect estimates that are outliers and removes them from the analysis by regressing the variant-outcome associations on variant-exposure associations. A global heterogeneity test is then implemented to compare the observed distance between residual sums of squares of all variants to the regression line with the distance expected under the null hypothesis of no pleiotropy [41]. Furthermore, MR-Robust Adjusted Profile Score (RAPS) was applied. This method can correct for pleiotropy using robust-adjusted profile scores. We consider as results causal estimates that agreed in direction and magnitude across MR methods, passed nominal significance in IVW MR, and did not show evidence of bias from horizontal pleiotropy using heterogeneity tests. All analyses were done using the R software (version 3.4.2 R Core Team, 2017).

### Ethics

This investigation uses published or publicly available summary data. No original data were collected for this manuscript. Ethical approval for each of the studies included in the present analysis can be found in the original publications (including informed consent from each participant). The study conforms to the ethical guidelines of the 1975 Declaration of Helsinki.

## Results

Demographic characteristics of the study participants are shown in Online Resource 1. The genetic instruments and observed phenotypes are shown in Online Resource 2, and the instrument associations for blood lead levels are shown in Online Resource 3. The instruments have F-statistics higher than threshold, making significant bias from use of weak instruments unlikely [42]. The results, expressed as beta-coefficient for blood lead per 1 standard deviation (SD) increase in outcomes, are presented in Table 1.

**Table 1:** Results of the Mendelian randomisation (MR) analysis for effects of blood lead on CKD and eGFR

**Exposures**		**MR**				**Heterogeneity**			**Pleiotropy**		
		**Method**	**Beta**	**SE**	***p***	**Method**	***Q***	***p*-value**	**Intercept**	**SE**	***p***
**Blood lead**	**CKD**	**MR Egger**	0.2227	0.2405	0.397	**MR-Egger**	6.389	0.272	−**0.025**	**0.029**	**0.430**
		**WM**	−0.02288	0.07127	0.7482						
		**IVW**	0.02677	0.05943	0.6524	**IVW**	7.036	0.293			
		**RAPS**	0.02344	0.06442	0.716						
	**eGFR (overall)**	**MR Egger**	−0.01488	0.01406	0.3381	**MR-Egger**	6.642	0.248	** 0.0017**	**0.0017**	**0.377**
		**WM**	−0.00294	0.004129	0.4766						
		**IVW**	−0.00151	0.003539	0.6688	**IVW**	7.793	0.245			
		**RAPS**	−0.00197	0.003753	0.5989						
	**eGFR (No T2DM)**	**MR Egger**	−0.01441	0.01536	0.3913	**MR-Egger**	7.854	0.164	** 0.0019**	**0.0019**	**0.355**
		**WM**	−0.00098	0.004262	0.8183						
		**IVW**	0.000871	0.003896	0.8232	**IVW**	9.485	0.148			
		**RAPS**	0.000262	0.00407	0.9486						
	**eGFR (T2DM)**	**MR Egger**	0.07222	0.04948	0.2043	**MR-Egger**	2.113	0.832	−**0.013**	**0.006**	**0.096**
		**WM**	−0.03251	0.01742	0.06207						
		**IVW**	−0.03416	0.0138	0.01328	**IVW**	7.068	0.314			
		**RAPS**	−0.03816	0.01433	0.007746						

*WM* weighted median, *IVW* inverse variance weighted, *SE* standard error, beta beta-coefficients, *MR* Mendelian randomisation, *CKD* chronic kidney disease, *eGFR* estimated glomerular filtration rate, RAPS robust adjusted profile score, *T2DM* type 2 diabetes mellitus

**Fig. 1:**
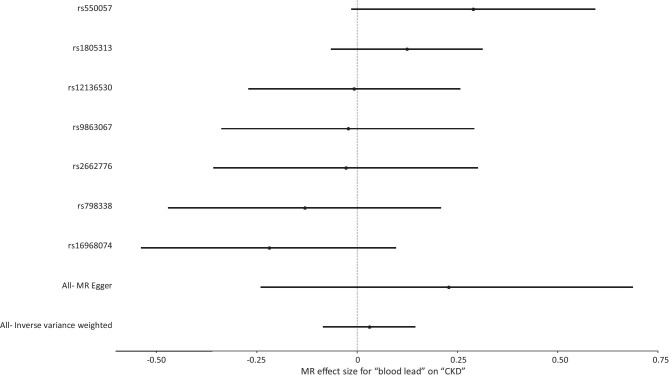
Forest plot of overall and individual SNP effects on CKD. SNP single nucleotide polymorphism; CKD chronic kidney disease

**Fig. 2:**
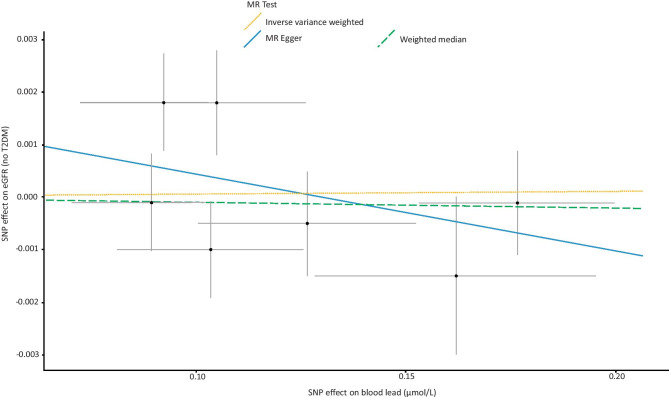
Scatter plot of the association of the effect of SNP-determined blood lead on eGFR in individuals without T2DM. Each black point represents an SNP, plotted by the estimate of SNP on blood lead level (x-axis, nmol/L) and the estimate of SNP on eGFR (y-axis, mL/min). The slopes of each line represent the potential causal associations for each method. SNP single nucleotide polymorphism; T2DM type 2 diabetes mellitus; eGFR estimated glomerular filtration rate

**Fig. 3:**
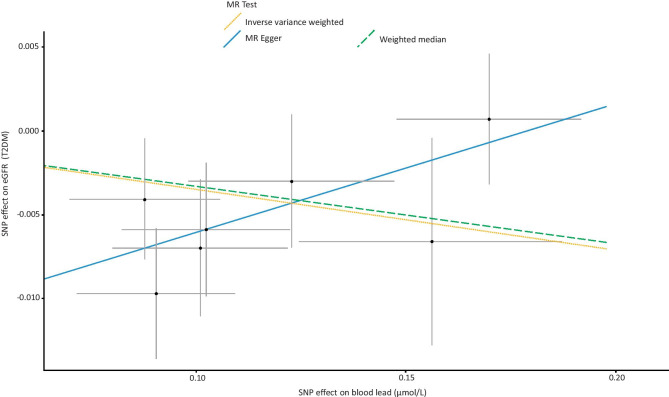
Scatter plot of the association of the effect of SNP-determined blood lead on eGFR in individuals with T2DM. Each black point represents an SNP, plotted by the estimate of SNP on blood lead level (*x*-axis, nmol/L) and the estimate of SNP on eGFR (y-axis, mL/min). The slopes of each line represent the potential causal associations for each method. SNP single nucleotide polymorphism; T2DM type 2 diabetes mellitus; eGFR estimated glomerular filtration rate

Genetically higher blood lead levels had no significant effect on risk of CKD (IVW = Beta: 0.02677, *p* = 0.652: Table 1; Fig. 1) or level of eGFR (IVW = Beta: −0.001514, *p* = 0.668, Table 1) in this sample. After grouping subjects based on type 2 diabetes status (no type 2 diabetes vs. type 2 diabetes), genetically determined levels of blood lead had no significant impact on subjects without type 2 diabetes (IVW = Beta: 0.0008706, *p* = 0.823: Table 1; Fig. 2). However, in subjects with type 2 diabetes, a significant effect on eGFR was observed (IVW = Beta: − 0.03416, *p* = 0.0132: Table 1; Fig. 3).

Heterogeneity results and pleiotropy bias are also shown in Table 1. Estimation based on both MR Egger and IVW was higher than 0.05, which indicated no chance of heterogeneity (all IVW *p* > 0.158, all MR Egger *p* > 0.175). Further, the results of the MR-PRESSO did not indicate any outliers for all the estimates. The horizontal pleiotropy test, with very negligible Egger regression intercept, also indicated a low likelihood of pleiotropy for all of our estimations (all *p* > 0.139). The results of the MR-RAPS were identical with the IVW estimates, highlighting again a low likelihood of pleiotropy. The results of the leave-one-out method demonstrated that the links were not driven by single SNPs.

## Discussion

In this study, we have analysed a set of genetic variants that were demonstrated to be associated with blood lead levels in order to determine their relationship with renal function. Mendelian randomisation analyses showed that higher blood lead might be linked with less favourable renal function but only amongst individuals with type 2 diabetes.

Lead is commonly used for industrial purposes, and chronic exposure to lead, either through industrial or environmental means, has been responsible for numerous cases of lead toxicity or plumbism [43–45]. Concerns over the toxicity of lead have led to the phasing out of some of its use in industry and consumer goods [46–48]. In particular, lead in petrol and paint is believed to have been one of the principle contributors to increased blood lead levels in humans and was phased out of use in the USA from the late 1970s [49].

While the relation between lead exposure and CVD is well established [50, 51], the role of blood lead levels in the development of CKD and reduced renal function remains controversial. Indeed, cross-sectional studies of lead-exposed workers often report changes in markers of kidney function, such as increases in creatinine clearance, without clinically significant reductions in eGFR or diagnosis of renal failure [28, 29, 52, 53]. In a sample of 803 Korean lead workers, blood lead levels were significantly associated with increased uric acid (UA) levels (which is known to be nephrotoxic) in the oldest tertile of workers with serum creatinine greater than the median [28]. Similarly, in a sample of 229 Chinese lead battery factory workers, there was an increasing trend in the dose–response relationship between blood lead levels and indicators of renal function of blood-urea nitrogen (BUN) and UA [29]. However, only those with longer periods of occupational lead exposure had a higher possibility of reduced renal function. Cardenas et al. [52] compared data from 50 Belgian, lead-exposed workers with age-matched controls and reported no indication of significantly increased proteinuria in those exposed to lead. However, blood lead was associated with altered urinary excretion of 6-keto-PGF and thromboxane, eicosanoids which may contribute to the pathologies involved in renal failure and hypertension [54]. Pollock and Ibels [53] presented a case study of 6 men exposed to lead from paint in Australia and suffering from lead intoxication. While some measures related to renal function, such as serum uric acid, urinary protein and creatinine clearance, were abnormal in some cases, these were not consistently observed in the majority of the cases presented. Thus, it can be seen that while lead exposure may have effects on renal-related parameters, a conclusive relationship between lead and CKD in otherwise healthy populations cannot be drawn. Furthermore, such cross-sectional data is not sufficient to determine a causal relationship between lead exposure and CKD, and thus, sufficiently controlled, longitudinal studies as well as mechanistic evidence for a causal effect would be needed. However, the use of MR analysis can overcome the limitations of observational studies as MR is a powerful tool for the detection of causation [34]. As such, the results of this study provide evidence that small, life-long changes in genetically determined blood lead do not impact the development of CKD in individuals without type 2 diabetes.

As such, our study did find an association between genetically determined blood lead and decreased eGFR in those presenting with type 2 diabetes. Renal tubule damage is a common feature of type 2 diabetes and is considered to be a pathway to glomerular dysfunction associated with proteinuria and the development of CKD in those with type 2 diabetes [55]. It could be speculated that the nephrotoxic effects of substances such as UA, which are elevated in lead-exposed individuals, might contribute to the development of diminished kidney function in those already experiencing renal tubule damage due to type 2 diabetes [28, 29]. This might explain why high blood lead is only seen to contribute to CKD in those with type 2 diabetes, i.e. those with pre-existing damage to renal tubules.

We believe this to be the first study to report that there is a relationship between genetically determined blood lead levels and reduced eGFR in individuals with diabetes. Indeed, diabetes is a frequent comorbidity in CKD and is believed to contribute to the development of impaired renal function [7, 20]. It has been observed that individuals with earlier onset type 2 diabetes, and consequently longer duration of diabetes, have a 2.6-fold higher risk of CKD, compared to those with later-onset diabetes [56]. Lead is known to contribute to oxidative stress in those exposed to high levels [57, 58], and more specifically, lead has been reported to lead to oxidative stress and apoptosis in in vitro human mesangial cells which may be a possible mechanism for lead-induced nephrotoxicity [59]. Similarly, lead exposure is known to affect the immune system resulting in altered cytokine metabolism and a proinflammatory response [60]. We propose that as the diabetic state is associated with metabolic derangement such as elevated oxidative stress [61] as well as elevated levels of proinflammatory cytokines [62, 63] and renal tubule damage [55], lead exposure may accelerate and augment these detrimental processes (which may not be present in those without diabetes) and lead to renal dysfunction more readily in subjects with type 2 diabetes. Further research is needed to investigate the mechanisms of the blood lead–related renal dysfunction amongst those with diabetes.

A major strength of our study is the large sample population with access to individual participant data of high validity for eGFR and CKD status, and with the relevant SNPs available for blood lead concentration. Additionally, the use of MR methods allows us to examine the potential causal effects of blood lead, largely without the disadvantages of confounding or reverse causation. We checked for known pleiotropy using Ensembl and found few known phenotypes of the genetic predictors of blood lead apart from multiple associations for rs550057 (ABO) (Online Resource 2). A potential limitation of this study is the use of a predominantly white, ethnically European population which limits the generalizability of the results. As such, ethnically diverse GWAS and MR studies are necessary to generalize MR results to people of different ancestries. Furthermore, while this MR analysis provides evidence on the effect of smaller life-long, genetically determined blood lead levels, it may not necessarily apply to short-term larger changes in blood lead, due to environmental factors. Another potential concern with MR analysis is the risk of stratification bias, which would only be an issue if type 2 diabetes resulted from both elevated blood lead levels and the presence of CKD. Finally, due to the limited number of shared SNPs identified by both the QIMR and ALSPAC studies (*n* = 3) (Online Resources 4 and 5), it is not possible to perform a sensitivity analysis to determine differences between the results of both datasets. As such, future research should endeavour to perform such sensitivity analyses, as sufficient data on relevant SNPs becomes available.

In conclusion, this investigation found evidence to support a potential causal association between genetically determined blood lead levels on renal function in individuals with type 2 diabetes. However, in subjects without diabetes, no such causal relationship was identified. While further investigation is required to investigate the link between lead exposure and indices of renal function in those with diabetes, this novel data also contributes to the current understanding that the relationship between lead exposure and CKD in non-diabetic individuals may simply be associative.

## Supplementary Information

Below is the link to the electronic supplementary material.

Supplementary file1 (DOCX 99840 KB)

## Data Availability

The datasets analysed in this study are publicly available summary statistics.

## Declarations

### Disclaimer

The study funder was not involved in the design of the study; the collection, analysis, and interpretation of data; writing the report; and did not impose any restrictions regarding the publication of the report.
