# Supplementary material for: Genetically determined blood lead is associated with reduced renal function amongst individuals with type 2 diabetes mellitus: insight from Mendelian Randomisation
Source: J Mol Med (Berl). 2021 Oct 18;100(1):125–34. doi: 10.1007/s00109-021-02152-5 (PMC8724171; doi:10.1007/s00109-021-02152-5)
Supplement: Supplementary file 1 — Supplementary file1 (DOCX 99840 KB) [file 109_2021_2152_MOESM1_ESM.docx]

**Genetically determined blood lead is associated with reduced renal function amongst individuals with type 2 diabetes mellitus: Insight from Mendelian Randomisation**

**Journal**

Journal of Molecular Medicine

**Authors**

Mohsen Mazidi, Department of Twin Research and Genetic Epidemiology, King's College London, London, UK. <https://orcid.org/0000-0003-3059-229X>

Richard Kirwan, School of Biological and Environmental Sciences, Liverpool John Moores University, Liverpool, UK. <https://orcid.org/0000-0003-4645-0077>

Dr Ian G. Davies, Research Institute of Sport and Exercise Science, Liverpool John Moores University, Liverpool, UK. <https://orcid.org/0000-0003-3722-8466>

**Corresponding author:** Richard Kirwan, r.p.kirwan@2018.ljmu.ac.uk

**Online Resource 1.** Demographic characteristics of study participants for the UK (ALSPAC) studies and Australian (QIMR).

| **Study** | **ALSPAC** | **QIMR** | |
| --- | --- | --- | --- |
| N with phenotype and genotype data | 2830 | 1570 | 1104 |
| Sex (percent Female) | 100% (all pregnant at time of blood collection) | 66% | 50% |
| Age (mean ± SD) | 28.4 ± 4.8 | 46.0 ± 11.8 | 49.0 ± 13.0 |
| Smoking status (percent current smokers) | 32% | 20% | 27% |

QIMR: Queensland Institute of Medical Research; ALSPAC Avon Longitudinal Study of Parents and Children

Adapted from Warrington et al (2015) [34]

**Online Resource 2.** Identified SNPs and observed phenotype.

| **SNP** | **Phenotype** | **PMID** |
| --- | --- | --- |
| rs12136530 | Blood lead levels | 25820613 |
| rs2662776 | Blood lead levels | 25820613 |
| rs76153987 | Blood lead levels | 25820613 |
| rs9863067 | Blood lead levels | 25820613 |
| rs79019069 | Blood lead levels | 25820613 |
| rs116864947 | Blood lead levels | 25820613 |
| rs6462018 | Blood lead levels | 25820613 |
| rs798338 | Blood lead levels | 25820613 |
| rs60580184 | Blood lead levels | 25820613 |
| rs1805313 | Blood lead levels | 25820613 |
| rs550057 | Blood protein levels | 29875488 |
|  | LDL cholesterol | 25961943 |
|  | Estimated glomerular filtration rate | 31015462 |
|  | Total cholesterol levels; Low density lipoprotein cholesterol levels | 29507422 |
|  | Allergic disease (asthma, hay fever or eczema) | 29679657 |
|  | Lung function; Red blood cell count | 30595370 |
|  | Inflammatory biomarkers multivariate analysis | 33110245 |
|  | Cytokine network levels multivariate analysis | 31679650 |
|  | Hematocrit; Red blood cell count; Hemoglobin concentration | 32888493 |
|  | Total cholesterol levels; Low density lipoprotein cholesterol levels | 31217584 |
| rs144653651 | Blood lead levels | 25820613 |
| rs16968074 | Blood lead levels | 25820613 |

SNP: single nucleotide polymorphism; PMID: Pubmed identifier

**Online Resource 3.** Summary results of the genetic loci of our exposures

| **Traits** | **Gene name** | **SNP** | **beta** | **SE** | **EA** | **OA** | **EAF** |
| --- | --- | --- | --- | --- | --- | --- | --- |
| Blood lead | CAPZB | rs12136530 | -0.1265 | 0.026 | A | G | 0.198 |
|  | RGS5 | rs2662776 | -0.0894 | 0.019 | A | G | 0.529 |
|  | SRGAP3 | rs76153987 | 0.2381 | 0.0514 | T | C | 0.9516 |
|  | GBE1-CADM2 | rs9863067 | -0.1619 | 0.0335 | C | G | 0.0921 |
|  | AGTR1/CPB1 | rs79019069 | -0.3254 | 0.0689 | A | G | 0.0257 |
|  | THSD7A | rs116864947 | 0.4121 | 0.0805 | T | C | 0.0164 |
|  | EVX1-HIBADH | rs6462018 | -0.0918 | 0.02 | A | G | 0.5087 |
|  | MAGI2 | rs798338 | 0.0923 | 0.02 | A | C | 0.6433 |
|  | TTC26 | rs60580184 | -0.3215 | 0.0611 | A | G | 0.9742 |
|  | ABO | rs1805313 | -0.1764 | 0.0233 | A | G | 0.3051 |
|  | ALAD | rs550057 | 0.1035 | 0.0222 | T | C | 0.7461 |
|  | PTPN2-SEH1L | rs144653651 | -0.2584 | 0.0495 | A | G | 0.9283 |
|  | PEPD | rs16968074 | 0.1049 | 0.0213 | A | G | 0.3025 |

SNP: single nucleotide polymorphism; Beta: beta-coefficients; EA: effect allele; OA: other allele; EAF: effect allele frequency; SE: standard error

2

**Online Resource 4.** Genome-wide loci from QIMR data, showing the SNP with smallest P-value from each locus.

| **SNP** | **Freq A1** | **beta** | **SE** |
| --- | --- | --- | --- |
| rs1805313 | 0.726 | 0.265 | 0.044 |
| rs2662776 | 0.475 | 0.116 | 0.028 |
| rs76153987 | 0.044 | −0.350 | 0.097 |
| rs79019069 | 0.974 | 0.408 | 0.112 |
| rs6462018 | 0.515 | −0.101 | 0.029 |
| rs60580184 | 0.026 | 0.299 | 0.09 |
| rs16968074 | 0.711 | −0.110 | 0.032 |

QIMR: Queensland Institute of Medical Research; SNP: single nucleotide polymorphism; Beta: beta-coefficients; SE: standard error; Freq A1: frequency of the effect allele

Adapted from Warrington et al (2015) [34]

**Online Resource 5.** Genome-wide loci from ALSPAC data, showing the SNP with smallest P-value from each locus.

| **SNP** | **Freq A1** | **beta** | **SE** |
| --- | --- | --- | --- |
| rs1805313 | 0.726 | 0.265 | 0.044 |
| rs2662776 | 0.475 | 0.116 | 0.028 |
| rs76153987 | 0.044 | −0.350 | 0.097 |
| rs79019069 | 0.974 | 0.408 | 0.112 |
| rs6462018 | 0.515 | −0.101 | 0.029 |
| rs60580184 | 0.026 | 0.299 | 0.09 |
| rs16968074 | 0.711 | −0.110 | 0.032 |

ALSPAC Avon Longitudinal Study of Parents and Children; SNP: single nucleotide polymorphism; Beta: beta-coefficients; SE: standard error; Freq A1: frequency of the effect allele

Adapted from Warrington et al (2015) [34]
